# Application of Tribology Concept in Dental Composites Field: A Scoping Review

**DOI:** 10.3390/jfb13040287

**Published:** 2022-12-08

**Authors:** Giusy Rita Maria La Rosa, Luigi Generali, Calogero Bugea, Hani F. Ounsi, Gergely Benyőcs, Prasanna Neelakantan, Eugenio Pedullà

**Affiliations:** 1 Department of General Surgery and Medical Surgical Specialties, University of Catania, 95123 Catania, Italy; 2 Department of Surgery, Medicine, Dentistry and Morphological Sciences with Transplant Surgery, Oncology and Regenerative Medicine Relevance, University of Modena and Reggio Emilia, 41125 Modena, Italy; 3 Independent Researcher, 72100 Brindisi, Italy; 4 Department of Endodontics, Faculty of Dental Medicine, Lebanese University, Beirut 1533, Lebanon; 5 Private Practitioner, Precedent Dental Office, 1088 Budapest, Hungary; 6 Discipline of Endodontology, Faculty of Dentistry, The University of Hong Kong, Hong Kong SAR, China

**Keywords:** biotribology, friction, dental resin composites, lubrication, scoping review, tribology, wear

## Abstract

Tribology is the discipline concerning the application of friction, lubrication, and wear concepts of interacting surfaces in relative motion. A growing interest has developed in tribology application in medical biomaterials, such as resin composites used in restorative dentistry. Yet, the keywords “tribology” and “biotribology” are little applied in the pertinent publications. The aim of this scoping review was to offer an overview of tribology application in dental composites research and to identify knowledge gaps and address future research. A literature search was conducted on Pubmed and Scopus databases and the studies investigating the tribological behavior of resin composites were included for qualitative synthesis. The majority of studies on dental tribology were published in the research areas of mechanical engineering/nanotechnology and differed in several methodological aspects. The preponderant engineering approach and the lack of standardized testing make the laboratory findings poorly informative for clinicians. Future research should focus on the tribological behavior of dental materials composites by means of an integrated approach, i.e., engineering and clinical, for improving development and advancement in this field of research.

## 1. Introduction

Tribology is the science of friction, lubrication, and wear applied on interacting surfaces in relative motion [1]. Friction is defined as the rubbing of an object against another [2]. It can be minimized by the application of substances able to make smooth the contact surfaces [3]. Wear is the gradual removal of material from surfaces as the result of mechanical or chemical injuries [3]. The concept of tribology has become popular in recent years, mainly in its relationship with biology, which is known as biotribology. The use of medical biomaterials in dentistry represents one of the most promising applications of the biotribology concept [4,5].

Human teeth act as a tribological system being subject to friction, lubrication, and wear phenomena during masticatory and phonetic functions. Furthermore, dental surfaces undergo degradation processes including trauma, caries, and tooth tissue loss, often accelerated by ageing [6,7]. Thus, biocompatible materials and nontoxic materials have been proposed and developed to replace the dental tissue damaged. Dental composites consist of an inorganic filler (e.g., borosilicate glass, alumina, hydroxyapatite, and zirconia) immersed in a polymer resin matrix (e.g., bisphenol to glycidyl methacrylate (Bis-GMA) and triethylene glycol dimethacrylate (TEGDMA)) [8,9]. A coupling agent, typically a silane, enhances and stabilizes the chemical bond between the filler and the resin matrix [10,11]. Composites are commonly used in restorative dentistry because of several benefits such as suitable mechanical behavior and good aesthetics. Yet, they suffer the same phenomena of physical and chemical wear described for dental issues [12,13,14].

To understand the wear pattern between the dental tissues and restorative materials, the oral cavity is compared to a tribological model constituted of four elements, i.e., solid, counter body, interfacial, and environmental elements. The first is the solid object represented by the tooth; the second can be a solid, liquid, or gas and, within dental model, is comparable to the opposite tooth; the third is frequently a solid or a liquid, such as the food bolus or saliva, respectively; finally, the air is the environmental element in which wear process take place [6].

According to the contact material, two different patterns of abrasive wear are described: two- and three-body abrasion [15,16]. The former is due to the friction generated by two moving solids when their surfaces are in direct contact. It is common in hard but fragile materials such as ceramics [15,17]. The latter occurs when solids are in mutual motion and abrasive particles interpose between them [18]. The generalized or local occurrence of this mechanism has been investigated in the oral cavity. When generalized, it is commonly linked with the abrasive effects of alimentary particles during the chewing activity [19].

Tooth wear and consequent resin degradation are relevant clinical issues particularly now that the average age of populations has risen significantly. Understanding the abrasion, lubrication, and wear phenomena in dental apparatus by the application of tribology could be extremely helpful in reducing causal factors and developing new high-performance dental restorative materials [20,21,22].

For all these reasons, current research, mainly in conservative dentistry, is oriented towards tribology even if often in an incomplete way. Indeed, the wear phenomenon is investigated singularly and not in a mutual relationship with friction and lubrication concepts. Preliminary research using the keywords “dentistry” and “tribology” retrieved only 60 results on Pubmed (4 October 2022) suggesting that the terms are poor applied in dentistry field.

The aim of this scoping review was to offer an overview on tribology application in dental composites research in order to identify knowledge gaps and address future research needs.

## 2. Materials and Methods

This scoping review followed as closely as possible the Preferred Reporting Items for Systematic Reviews and Meta-Analyses (PRISMA) Extension for Scoping Reviews [23] and focused on the following research question: “What is the application of tribology concept in dental resin composites research?”

### 2.1. Inclusion Criteria

In vitro animal and human studies that evaluated the application of tribology in dental composites materials were included. Review articles, conference reports, short communications, and editorials were excluded. No language or date restrictions were applied.

### 2.2. Search Strategy

The literature search was performed on 23 September 2022. The search was carried out by two independent reviewers on PubMed and Scopus databases. The strategy was adopted on database, applying the MESH terms and the keyword identified in the most relevant studies: (tribology OR biotribology OR (“dental wear” AND “dental friction” AND lubrication)) AND (“dental material*” OR “dental composite*” OR “dental resin*” OR “conservative dentistry”). Included articles and reviews were further screened for other potentially relevant articles. All of the review process was conducted using the EndNote program (EndNote X9; Thomson Reuters, New York, NY, USA). Articles were screened in titles and abstracts by two independent reviewers to ensure inclusion criteria were satisfied. All duplicates were removed.

### 2.3. Study Selection

Studies with insufficient information were temporarily included and successively screened independently in the full text by the two same reviewers. Any disagreement about the inclusion was solved by discussion. No intervention of a third examiner was necessary. Articles satisfying the inclusion criteria were considered for the qualitative synthesis.

### 2.4. Data Extraction and Synthesis

The relevant data were extracted by using a standardized form developed for this purpose. The following items were recorded and tabulated: author and year, journal, sample size (n), study design, objective, methodology, and main findings. The qualitative synthesis focused on the principal tribology applications in dental composites, with particular reference on the tested materials and properties investigated.

## 3. Results

### Study Selection

**Figure 1:**
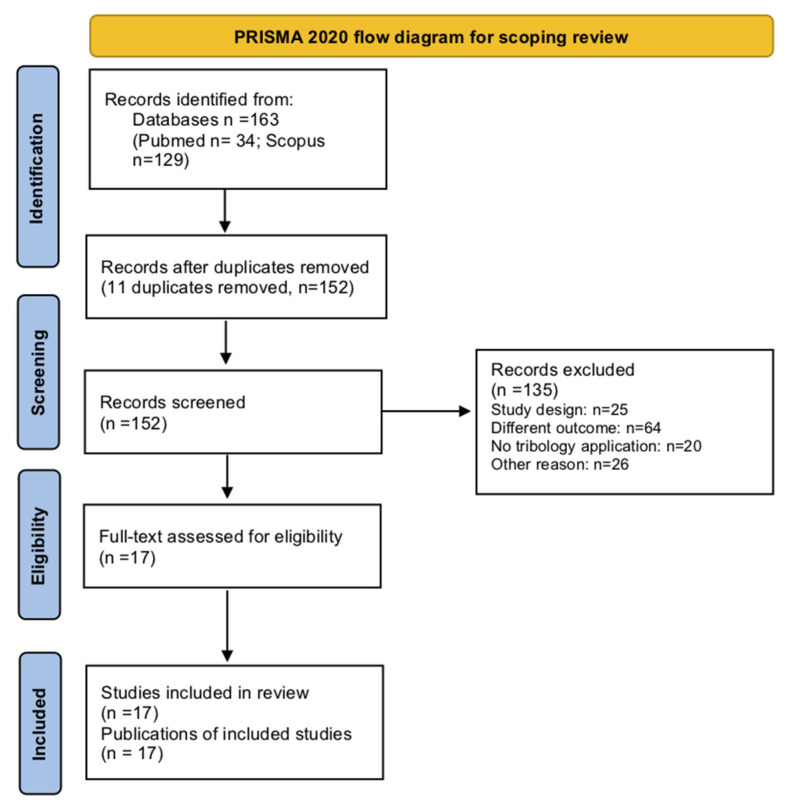
Flow chart of review process.

The flow chart for study selection according to PRISMA 2020 is reported in Figure 1. The search identified 163 potentially relevant studies (PubMed: n = 34; Scopus: n = 129). After removal of duplicates (n = 11) and articles not fulfilling the inclusion criteria (n = 135), 17 laboratory studies met the eligibility criteria for qualitative synthesis. No animal or human studies were identified. The included studies are listed in the Table 1 [22,24,25,26,27,28,29,30,31,32,33,34,35,36,37,38,39].

The number of publications increased through the years and 13 of 17 articles were published after 2010. In addition, 13 studies were published in journals that covered research into mechanical engineering or technology of nanosized and nanostructured materials, including advanced ceramic materials and reinforced plastics and polymer composites [25,26,27,28,29,30,31,32,33,35,36,37,38]; three studies were published in journals that focused on the application of biomaterials and tissue engineering in the medical sciences [24,34,39] and only one was published in a dentistry journal [22].

Eight studies investigated the effect of the size, concentration, and nature of powder- filling particles on the tribological behavior of resin composites [22,24,30,31,33,35,37,38]. Two articles assessed the influence of exposure to different acid solutions [27,29] and two assessed the effect of different pH and aging times [25,36]. Other studies evaluated the effect of temperature and immersion in beverages [26], thermocycling [28], smokeless tobacco at different aging times [34], and different light curing units [39] on mechanical behavior of dental resins. Finally, one article compared the tribological behavior of human enamel with that of some resin composites [32].

The tribological behavior was investigated for volume loss/penetration depth [22,25,26,29,30,32,33,34,35,39], roughness [27,28,31,39], plasticity index [26], coefficient of friction [24,25,27,28,30,33,36], wear rate [24,27,33,36,37,38], Vickers hardness [24,26,27,28,29,31,36,39], elastic modulus [28,29,35], abrasion resistance [35], flexural [28], and compressive [33] strength.

**Figure 2:**
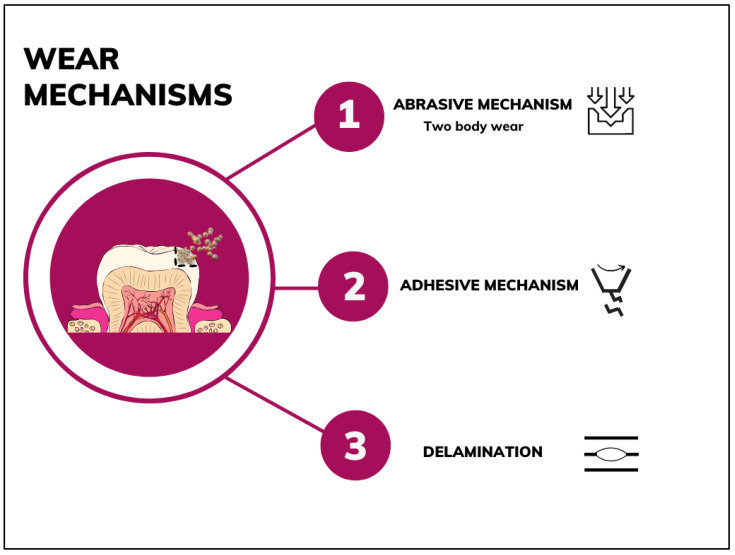
The main wear mechanisms identified in the included studies.

Several macroscopic and microscopic techniques were employed to investigate tribological and mechanical properties of tested materials. Thirteen studies used tribological testing [22,24,25,27,30,32,33,34,35,36,37,38,39] and ten quantitative assessment methods such as profilometer [22,39] or surface hardness or nanoindentation techniques [24,26,27,28,29,31,35,36,39]. Qualitative evaluation included scanning electron microscopy (SEM) [22,25,29,31,32,33,34,35,36,37,38,39], atomic-force microscopy (AFM) [26,27,31], energy-dispersive X-ray spectroscopy (EDS) [22,24,32,37,38], scanning probe microscopy (SPM) [29], optical microscopy [33,36], and induced coupled plasma optical emission spectroscopy [35]. The main wear mechanisms are illustrated in Figure 2.

Overall, tribological behavior of dental-resin-based composites was affected by the morphology, particle uniformity, and concentration of inorganic fillers [22,24,25,26,27,28,29,30,31,32,33,34,35,36,37,38,39]. More explicitly, Akhtar et al. tested how the addition of hydroxyapatite (HA) particles modified the tribological behavior of resin-based composites. The authors stated that the fabricated composite with 0.4 Wt% concentration of the cubic-shaped filler particles exhibited maximum hardness and reduced wear and COF values [24]. In addition to HA particles, Vargas et al. [35] also tested the influence of polymeric matric reinforcement with two different types of ceramic particles, alumina and silica; they found that the alumina hardness improved the abrasion resistance. Moreover, in the studies of Yadav et al. [37,38], the influence of a low amount (i.e., 0, 2, 4, 6, and 8 wt%) of nHA and aluminum or titanium oxidum [37] and Ha-zinc oxide filling (from 0% to 8%) [38] on the tribological behavior of dental resin composites was tested. The authors concluded that load, time, and filler significantly impacted the wear rate of tested composites [37,38]. According to Mystkowska & Dąbrowski [30], the organic fillers composites exhibited the lowest COF and wear. On the other hand, Souza et al. [33] found that resin with the highest inorganic filler content reported the lowest COF and wear rate. Furthermore, as reported by Rodríguez & Casanova [31], roughness of dental composite was affected by particle size while the nanohardness was affected by the concentration of the reinforcement materials. In particular, the authors showed that silica nanoparticles presented lower values of roughness compared with silica and silica–zirconia nanoclusters while no significant differences emerged for hardness.

Altaie et al. [22] determined the wear behavior of differently filled resin-based composites. Although all composites showed abrasive wear to a variable degree, the universal nano-filled composite Filtek Supreme (3M ESPE) exhibited the major wear volumetric loss compared with the other composites.

In addition, nano and nanohybrid composite samples also demonstrated suitable properties when the samples were thermocycled and immersed in tea and soft drinks [26] or exposed to acid environment [27,29]. Antunes & Ramalho [25] showed that the 22-month period and pH 9 were the most impactful conditions on wear pattern of the tested composites. Similarly, Wu et al. [36] outlined that the most extreme pH values (i.e., 1 and 13) and prolonged aging had a detrimental effect on mechanical and wear properties of tested composites. Moreover, the hybrid composites with high filler loading and distribution exhibited better mechanical and wear indices than nano-filled composites with low filler. According to Carreira et al. [28], thermocycling had no influence on hardness of nanohybrid and microfilled resin-based composites while decreased the elastic modulus and COF of those microfilled.

Topcu et al. [39] assessed the effect of different light curing units (i.e., light-emitting diode, LED, and quartz-tungsten-halogen, QTH) on the mechanical behavior of some resin composites. The findings revealed that the type of light unit influenced the microhardness of resin composites tested, with higher values for QTH light units. In addition, the nano-filled composite (i.e., Clearfil Majesty^TM^ Posterior, Kuraray) exhibited higher microhardness, less surface roughness, and higher wear resistance when compared with the other tested composites, regardless the light curing unit.

Suryawanshi & Behera [34] explored the effect of smokeless tobacco on the tribological properties of two dental resin composites. Interestingly, the wear depth intensity was negatively affected by immersion in tobacco solution.

Sajewicz [32] was the only researcher who compared the wear resistance of tooth enamel with commercial resin-based composites, by means of energy approach. Wear resistance, correlated with the dissipated frictional energy, was significantly higher for tooth enamel compared with the investigated composites [32].

## 4. Discussion

Despite the advantages of dental composites [40,41,42], wear of resin composites represents one of the main issues associated with their clinical use [43,44]. The shape, size, and distribution of the filler particles as well as the weight and volume percentage of the inorganic filler are only some of the factors that affect the wear of resin materials [40,45,46]. Temperature and environmental conditions such as pH and lubrication level are other conditions able to significantly impact the clinical performance and durability of composites [47,48,49,50].

For all these reasons, manufacturers have introduced materials with enhanced physical and mechanical properties [9]. An extensive knowledge of the mechanical behavior of composites is required to know which are property-enhancing without damaging other properties. Within this context, the application of the “tribology” concept to research on composite dental materials can be extremely useful for a better understanding of the mechanical behavior of composites [51,52]. The application of tribology in the dental field is justified by the fact that the oral cavity is comparable to a tribological system. The relative motion between teeth and restorative materials in the presence of saliva and external components such as food and drink results in contact and subsequent wear of teeth and restorative materials [6,51]. Understanding the tribological mechanisms underlying tooth wear is important to minimize dental resin damage and promote efficient development of new biomaterials.

A scoping review is a flexible approach for reviewing the existing literature on a topic in order to identify gaps in knowledge and conceptual boundaries and direct future research [53,54]. Thus, this methodological strategy is particularly indicated when a research topic has not been extensively investigated [55]. The primary aim of our review was exploratory in order to assess whether the tribology concept has been currently applied in the research field of dental composites. Despite a huge number of studies on wear phenomena of dental composites having been published, few of them have applied a tribological methodological approach. Only one of seventeen studies identified was published in a dental journal, confirming the trend observed in the exploratory search which showed that the keywords “tribology” and “biotribology” are poorly applied in dentistry, and even then, mainly in the conservative field.

Although the most reliable way to determine the clinical performance of dental composites would be to conduct in vivo testing, some methodological limitations make them inadequate for providing standardized and replicable results [51]. Consequently, in vitro testing is much more used in research and results are more informative because of the precise control of variables involved [51]. More specifically, the experimental parameters, such as the normal load (e.g., 1–100 N and up to 1000 N in bruxism), number of cycles (e.g., 5000–120,000 cycles), test frequency (e.g., 1.2–1.7 Hz), and lubrication medium are adapted to the type of abrasion mechanism to be simulated [56]. Despite the rigorous control of above variables, in vitro tests cannot reproduce the complexity of the oral environment. Therefore, they are useful for obtaining insights into wear patterns but cannot be directly translated to the clinics [57]. Table 1 provides a summary of the main testing used in the included studies. In most of the studies, the contact was simulated by a two-body wear test in pin-on-flat or ball-on-flat models [22,24,25,27,30,32,33,34,35,36,37,38,39]; thus, the geometries required for conducting the tests were a flat sample and a cylindrical pin or sphere [6]. The reciprocating tribometer is one of the most widely used models. It consists of a cylindrical pin or sphere, in contact with a flat sample, subjected to a contact pressure generated by a vertical load. The apparatus moves along a linear trajectory alternately forward and backward, at imposed frequency parameters [6]. The tribometer provides the coefficient of kinetic friction which can be considered an index of material wear [58,59]. The limitation is that the reciprocating tribometer allows only alternating sliding and not impact to be simulated [6]. The advantages include the standardized conditions and ability to conduct the test under lubricated conditions [6].

Simulating lubrication represents a fundamental aspect in tribological investigation. Six of the 17 included studies mentioned that the test was conducted under lubricated conditions [24,30,32,33,34,36]. Lubrication is reproduced either by immersion in a bath or by pumps that continuously lubricate the contact area during the test [6]. In vitro lubrication can only be simulated by artificial saliva since natural saliva cannot ensure standardized pH conditions [60]. Studies performed under dry conditions should be interpreted with caution because the composite material loss may be less in the wet environment due to the lubricating action of artificial saliva or similar lubricant [24].

Dental wear is studied both quantitatively (i.e., depth and volume of wear) and qualitatively (i.e., microscopic and macroscopic techniques of surface topography) [61]. Therefore, several macroscopic and microscopic methods and techniques have been used to investigate the tribological behavior of composite materials [22,24,25,26,27,28,29,30,31,32,33,34,35,36,37,38,39]. Detailed analysis of the different techniques is beyond the scope of this review. What is important to emphasize here is that the best and most desirable approach for future studies is to integrate both quantitative and qualitative techniques for obtaining more useful information on the wear mechanism and the tribology of the system [22].

The dimensions and content of inorganic filler particles as well as the nature of organic matrix polymers significantly impact the wear of dental composites [33,40,62]. Specifically, the immersion of harder inorganic micro- and nano-particles in a matrix of lower hardness allowed the material’s resistance to abrasion to be improved. In addition, increase in inorganic filler volume/weight percentage enhanced the mechanical properties of dental resin [24,26,33,36,37]. Furthermore, the increase in inorganic content implies a reduction in the volume of the organic component. This mechanism decreases the polymerization contraction and thermal expansion coefficient of the resin [33,41]. Of note, the addition of the filler itself, regardless of the other properties, improves the tribological properties of the polymer matrix. This mechanism can be explained by the presence of grooves at the sliding contact. The addition of particles up to a certain optimal value fills the grooves making the contact surface flat and providing extra protection against wear [24,63]. Similarly, microhardness is enhanced with increasing filler up to a certain limit, beyond which microhardness is decreased. The composite has an optimal level of interfacial stress due to the added particles that provide a block to both extensional and shear deformation [24]. However, further increase in filling can induce voids to form and particles to agglomerate due to Van der Waal forces, resulting in reduced mechanical strength [64]. On the other hand, composites with organic friction modifiers (i.e., polyethylene, PE, and polytetrafluoroethylene, PTFE) exhibited the lowest friction coefficients when compared with composites with inorganic fillers [30]. This benefit could be linked to a better adhesion of organic fillers to the organic matrix which depended on filler grain forms and bonding forces [30]. The results are not directly comparable with the other studies due to differences in filler type and the value of loading applied on the sample.

Interestingly, roughness of dental composite was influenced by particle size while nanohardness was influenced by the concentration of the reinforcement materials [31]. This behavior justifies the application of nanoparticles to produce materials with lower roughness because the lower particle size, the smaller loss material [65].

As reported by Vargas et al. [35], the adding of different-sized particles of alumina and silica had a positive effect on Young’s modulus and wear resistance of resin-based dental composites. In addition, Yadav et al. [37,38] showed that nHA and aluminum or titanium oxidum [37] and Ha-zinc oxide filling (from 0% to 8%) [38] significantly improved the wear rate of tested composites [37,38]. The incorporating of ceramic particles in a polymeric matrix is a well-known mechanism to enhance the mechanical properties of the composites, depending on the hardness and size of particles [66,67,68]. Yet, the methodologies differ from others publications in several aspects such as in procedures for specimen preparation, nature/weight of particles added, and testing apparatus. Thus, the generalizability of results is limited and the applicability in clinical practice requires caution. Indeed, many other factors, including saliva, temperature, food particles, and muscle activity act simultaneously in real-life conditions [33,37]. Consequently, the wear rates could vary in different situations.

The environmental conditions have a notable impact on the in vivo degradation of dental resin composites. Expositions to liquids may determine the chemical degradation and wear of composites by two main mechanisms [69]. The first is linked to the diffusion of water molecules into the polymer network which resulted in plasticization and swelling of the polymer matrix [70]. The second is a hydrolysis reaction which degrades the siloxane bonds inducing the filler debonding [70].

These mechanisms are responsible for degradation or softening of resin-based composites which may impact their mechanical behavior including hardness, strength, and modulus of elasticity [69].

The magnitude of wear depth of resin-based composites was also negatively affected by prolonged immersion in a tobacco solution [34]. Even pH modifications affect mechanical behavior of resins with extreme pH values reported to negatively impact the wear resistance [25,36]. Indeed, an acid environment seems to induce hydrolysis of the resin matrix [71] while an alkaline environment promotes resin-to-filler debonding and fillers’ dissolution [72]. In addition, the tribological properties of resin materials such as dental composites are frequently influenced by temperature variations [26,28]. Overall, the sensitivity of dental composites to chemical and thermic degradation seems to be dependent on the type and volume of filler, the nature of monomers, and the degree of resin matrix cross-linking [25,26,27,29,36]. In addition, the mechanical behavior of resin composites, particularly surface microhardness, is influenced by the type and polymerization degree. Indeed, monomers not polymerized tend to decrease the hardness of inorganic filler and consequently composite hardness [39]. Moreover, the intensity, band width, and curing time of light units have been described as important parameters [73,74].

Of note, it is important to emphasize that human enamel possesses unique and not replicable tribological properties, mainly good wear resistance, which are determined by peculiar wear mechanisms not replicable in dental materials [32]. More specifically, as reported by Sajewicz [32], wear resistance, correlated with the dissipated frictional energy, was significantly higher for tooth enamel compared to the investigated composites [32]. Furthermore, the wear mechanism of the resin composites tested was characterized mainly by chemical modifications, while enamel was characterized by physical transformations. Sajewicz noted that energy approach is poorly applied in tribological investigations and this could explain why similar methodologies generate different findings [32].

When investigated, the preponderant wear mechanism was abrasive, determined by the contact between composite surface and antagonist, resulting in plastic deformation and material loss [22,25,27,28,29,30,33,34,35,37,38,39]. The abrasive pattern was also confirmed by the SEM analysis of wear facets with characteristic pitting, cracking, and material loss in variable degrees depending upon the particle size distribution, filler volume, and density. Furthermore, the presence of an adhesive mechanism [22,27] and delamination [28,29,36] were described. The former determined the material transferal from composite to the antagonist by cold welding through friction [75,76] and when combined with abrasive mechanisms, could be responsible for the increased wear-volume values reported for some resin-based composites [22]. The latter refers to the desquamation of reinforcement particles in sheets or flakes promoted by subsurface crack propagation along the sliding direction [77]. An exfoliation mechanism was also reported [24,36]. In this pattern, under the action of shear force, two adjacent layers were easily exfoliated with a consequent sliding movement of the layers, resulting in frictional and wear value reduction [64].

Of note, the type of wear process is strongly associated with testing apparatus [78]. In more detail, the two-body wear test replicates the attrition between opposite structures, while the three-body wear test mimics abrasion by food particles with multiple wear phenomena occuring simultaneously [79]. All included studies were conducted under two-body wear conditions.

Under the limitations of laboratory-based studies, some helpful strategies have been suggested to improve wear phenomena of resin-based composites. Nanofilled composites exhibited improved mechanical properties such as higher wear resistance [26,33,39] and lower roughness [31,39]. Hence, their application could be more beneficial than conventional composites mainly for long-lasting clinical restorations [26] and in acid environments such as those generated by exposition to acid beverages [29]. Yet, beneficial effects derived from variation in filler content and reinforcement are by no means automatic [35]. Indeed, the abrasive wear resistance of resin-based composites is material-dependent and cannot be deduced from their category, filler loading, or composite matrix [80]. In addition, the use of microcomposite resins requires extreme caution in patients with gastroesophageal reflux disease (GERD) disease which increases the hydrochloric (HCl) acid concentration in the oral environment [27].

Some limitations have to be considered. First, the review process. Secondary search and the quality assessment of the studies have not been conducted. Scoping reviews aim to provide an overview of available studies on a broad topic regardless of their bias assessment. Thus, a qualitative assessment of the methodological frame is not mandatory. In addition, the purpose of this scoping review was to explore the application of tribology and biotribology concepts in field of dental composites. Thus, the search strategy by keywords “tribology” and “biotribology” could have limited the findings retrieved. Yet, a comprehensive research on studies that assessed the wear phenomena of resin-based composites was beyond the aim of this review. Of note, the current review considered only the application of the tribology concept in the composites field. Many other fields of dentistry could be of interest for tribological applications. In particular, implantology possesses suitable characteristics to be treated under a tribological approach for improving the durability of prosthetic restorations and the implant survival rate in patients with systemic diseases [81,82].

A second limitation refers to the studies included. The studies differ in several methodological aspects including type of composite, properties investigated, testing apparatus, lubricant application, temperature, and pH values. Hence, the studies were not directly comparable. In addition, due to the complex nature of the oral environment, no methodological standard currently exists to investigate and measure mechanical and microstructural behavior of dental resin composites. Most of the current studies focus on comparison of wear characteristics of different composite materials; yet, studies for understanding the underlying mechanisms are poor and sparse [51]. In addition, wear behavior is often addressed from an engineering perspective without referring to clinical applications.

Based on the previous findings, future research should address three principal points:

1. A universal methodological standard applying the tribology concept is needed to standardize the studies conducted. More specifically, the wear behavior of composite materials should be investigated taking into account the reciprocal contact with dental tissues under dry and wet conditions.
2. Several aspects including the frictional force, mechanical energy dissipation, thermal micro- and nano-structural alterations have to be considered to fully understand the wear mechanism of dental composites.
3. An integrated approach, i.e., engineering and clinical, is needed to provide useful information for producers and translate the relevant finding to clinics. Understanding wear mechanism could assist the clinicians in selecting suitable materials mainly for patients with an increased wear risk.

## 5. Conclusions

This scoping review aimed to explore the application of the tribology concept in the research field of resin dental composites. The majority of studies on dental tribology were published in the area of research in mechanical engineering or nanotechnology and differ in terms of materials, testing apparatus, and environmental conditions. The preponderant engineering perspective and the lack of a standardized methodological approach make the laboratory findings poorly informative for clinicians. Further studies should focus on tribology behavior of dental materials composites, exploiting an integrated approach, i.e., engineering and clinical, for improving development and advancement in this field of research.

## Figures and Tables

**Table 1:** Main characteristics of the included studies.

Author & Year	Journal	Sample Size (n)	Study Design	Objective	Methodology	Main Findings
Akhtar et al. (2021) [24]	Journal of Materials Science: Materials in Medicine	NR	Laboratory study	To assess the tribological behavior: - wear resistance (wear rate mm^3^/Nm); - COF; - Vickers hardness (HV) of mono-dispersed HA particles and HA-reinforced acrylic resin (AR)-based composites.	EDS; pin-on-disk tribometer (lubricant used); microhardness tester.	Tribological behavior was influenced by the concentration and morphology of added particles. The fabricated composite with 0.4 Wt% concentration of the cubic-shaped filler particles exhibited maximum hardness and reduced wear and COF values.
Altaie et al. (2017) [22]	Journal of Dentistry	10	Laboratory study	To determine: - wear volume loss (mm^3^) and wear mechanism of universal microhybrid Filtek Silorane (3M ESPE); two universal nanofilled composites, Filtek Supreme (3M ESPE) and Clearfil Majesty Posterior (Kuraray); and three nanohybrids, Kalore (GC America), Venus Diamond (Heraeus Kulzer), and Tetric Ceram HB (Ivoclar-Vivadent) resin composites during short-term in vitro wear testing.	Modified pin-on-plate wear test apparatus; white light profilometer; SEM; EDS.	All composites showed abrasive wear in variable degree. The mean total wear volume of Filtek Supreme was significantly higher compared with the other resin composites. Filtek Supreme and Kalore reported signs of adhesive wear mechanism.
Antunes & Ramalho (2009) [25]	Wear	NR	Laboratory study	To determine how different values of pH (3,7, and 9) and aging time (3, 6, and 22 months) influenced tribological behavior in a reciprocating contact (wear volume, mm^3^ and COF) of Filtek P60 (3M ESPE), Prodigy Condensable Surefil (Kerr Dentsply), Synergy Compact (Coltène), Quixfil (Dentsply), CeramX (DeTrey Dentsply GmbH), and Alert (Jeneric-Pentron).	Reciprocating tribometer; SEM.	The 22-month period and the pH 9 were the most impactful conditions on wear pattern of the tested composites. The wear resistance of Surefil, Ceramx, Prodigy, and Quixfil increased after storage in buffer solution with pH 3. Conversely, Filtek, Alert, and Synergy showed an increase in the wear volume in the same conditions. Independently of pH values, aging damaged the interface matrix/reinforcement particles.
Ayatollahi et al. (2015) [26]	Materials Science and Engineering C	2 for thermocycling procedure; 5 for other tests	Laboratory study	To evaluate the effects of temperature change and immersion in two common beverages (tea and soft drinks) on the mechanical: - hardness (GPa); - elastic modulus (GPa); - plasticity index; - tribological properties: - wear resistance (penetration depth, ųm^3^) of universal Filtek Z250 (3M ESPE), nanohybrid Filtek Z250 XT, and nano Filtek Z350 XT dental resin composites.	Thermocycling apparatus; triboscope nanoindentation test system; AFM; nano-scratch test.	Overall, the tribological indicators of nano- and nanohybrid composites were less susceptible to temperature changes and immersion in beverages compared with universal.
Branco et al. (2019) [27]	Tribology Letters	32 (*n* = 4 for each group)	Laboratory study	To determine the effect of exposure to weak (lactic acid) and strong (hydrochloric acid) acids on: - roughness (nm); - hardness (HV); - friction and wear behavior (COF and wear rate) of a micro-hybrid methacrylate-based composite (Filtek Z250) after 1 and 7 days.	Microindentation tests; ball-on-plate nanotribological tests; AFM.	Both acids increased the surface roughness, mainly in samples in contact with hydrochloric acid for 1 day. Similar trend was recorded for the COF. The microhardness decreased after contact with both acids. The hydrochloric acid solution had a significantly negative impact on wear resistance of resin composite tested.
Carreira et al. (2017) [28]	Journal of the Brazilian Society of Mechanical Sciences and Engineering	120 (*n* = 20 for each group)	Laboratory study	To assess the effect of aging by thermocycling on the mechanical and tribological properties: - surface roughness; - dynamic and static elastic modulus (GPa); - flexural strength (MPa) and WOF (Jm^2^); - micro-hardness (MPa) and COF of nanohybrid (A) and microfilled (B) composites.	Surface profilometry; impulse excitation of vibration and four-point bending test; Vickers micro-indentation; scratch test.	Overall, composite A exhibited better mechanical properties than B. The thermocycling had no significant effect on hardness of both composites; conversely, it decreased the elastic modulus and COF of composite B. In general, composite B was more affected by thermocycling than A.
Fan et al. (2014) [29]	Journal of Nanomaterials	60 (*n* = 15 for each group)	Laboratory study	To assess the influence of different beverages (distilled water, orange juice, and Coca-Cola) on the surface mechanical properties: - nanohardness (GPa); - elastic modulus (GPa); - wear behavior (penetration depth, nm) of nanofilled Filtek Z350 (3M ESPE), nanohybrid TPH (Dentsply Caulk)^3^, microfilled Durafill (VS Heraeus Kulzer) and microhybrid Superlux (DMG Germany) resin composites.	Nanoindentation test; reciprocating nanoscratch test; SEM; SPM.	Acid beverages negatively impacted the mechanical and tribological behavior of microhybrid and microfilled composites. The nanofilled resin composites revealed the highest nanohardness/elastic modulus values and the least degradation.
Mystkowska & Dąbrowski (2009) [30]	Solid State Phenomena	NR	Laboratory study	To investigate the effect of organic fillers (PE, PTFE) and inorganic friction modifier (Si3N4, BN) on the tribological properties: - wear (volume loss, g) and COF of micro-filled dental composites.	Pin-on-disk tribotester (lubricant used)	The organic filler composites showed the lowest COF and wear. Increase in loading negatively impacted COF and wear of tested composites.
Rodríguez & Casanova (2018) [31]	Journal of Nanotechnology	Roughness: 4 cylinders for sample Nanohardness: 2 cylinders for sample	Laboratory study	To investigate the influence of nanoparticles and nanoclusters of silica and silica–zirconia on: - roughness (nm); - nanohardness (GPa) of dental resin composites.	AFM; nanoindentation; SEM; TEM.	Silica nanoparticles presented lower values of roughness in comparison with silica and silica–zirconia nanoclusters while no significant differences emerged for hardness. Overall, roughness seemed to be affected by particle size while nanohardness by the concentration of the reinforcement materials.
Sajewicz (2010) [32]	Journal of Engineering Tribology	48 samples from human teeth 48 (*n* = 12 for each composite)	Laboratory study	To compare wear resistance (volume loss) of (Arkona), Filtek (3M), Ful-Fil (Dentsply), and Ecusit (DMG) with that of human tooth enamel by an energy approach (J/mm^3^)	Customized tribometer (lubricant used); EDS; SEM.	Wear resistance, correlated with the dissipated frictional energy, was significantly higher for tooth enamel compared with composites. In addition, topographical analysis revealed that wear pattern was mainly due to physical changes in enamel, but chemical changes in the composites.
Souza et al. (2016) [33]	Tribology International	10 for each group	Laboratory study	To examine the abrasive and reciprocating sliding wear resistance (wear volume, mm^3^, wear rate, and COF) and compressive strength (MPa) of four dental resin composites: Grandio^R^ So (Voco, Germany); Ceram-X^TM^ (Dentsply, Germany); Clearfil^TM^ (Kuraray, Japan); and Natural Elegances ^R^ (Henry Schein, USA).	Universal testing machine (Instron 8874); TE-66 micro-scale abrasion equipment (stainless steel rotating ball); alumina ball and tribometer (lubricant used); optical microscope; SEM.	The resin with the highest inorganic filler content (Clearfil^TM^) showed the lowest COF and wear rate. Abrasion tests showed fine micro-scale abrasion as principal wear pattern while the reciprocating sliding tests revealed surface fatigue and abrasion.
Suryawanshi & Behera (2020) [34]	Proceedings of the Institution of Mechanical Engineers H: Journal of Engineering in Medicine	2 for each material	Laboratory study	To determine the effect of smokeless tobacco on the tribological properties (wear depth, ųm^3^) of two dental resin composites: Tetric N-Ceram (Ivoclar) and Z350 Dentin shade (3M ESPE) after 2, 3.5, 6, 15 days and 1 month.	Pin- on-disk tribometer (lubricant used); SEM.	Wear depth intensity was negatively affected by immersion in tobacco solution. Under different loading conditions, Z350 Dentin showed significantly less wear (and COF) compared with the Tetric N-Ceram in the presence of synthetic saliva, independently from tobacco immersion.
Topcu et al. (2010) [39]	Journal of Biomedical Materials Research Part B (Journal of Biomedical Materials Research)	10 for each composite	Laboratory study	To determine the effect of LED or a QTH light curing unit on mechanical behavior: - microhardness (VHN) - surface roughness (Ra) - wear resistance as wear loss (mg) of nanofilled ((Filtek Supreme (3M ESPE), Clearfil Majesty Posterior (Kuraray)), nanohybrid ((Ceram X (Dentsply deTrey GmbH), Premise (Kerr)), microhybrid ((Clearfil AP-X (Kuraray), Herculite XRV (Kerr)), minifilled hybrid ((Filtek Z250 (3M ESPE)), and hybrid (Quixfil (Dentsply deTrey GmbH)) composites.	Vickers hardness measuring instrument; profilometer; pin-on-disc tribometer; SEM.	The type of light unit influenced the microhardness of resin composites tested, with higher values for QTH. The nanofilled composite Clearfil MajestyTM Posterior showed higher microhardness, less surface roughness, and higher wear resistance in comparison with the other composites, regardless the light-curing unit.
Vargas et al. (2013) [35]	Materials Research Innovations	7 for each preparation	Laboratory test	To assess the mechanical (Young’s modulus, MPa) and tribological behavior (abrasion (mg/s)^−1^ and wear resistance as penetration depth, ųm^3^) of different dental materials filled with HA and reinforced with two different types of ceramic particles, alumina, and silica.	SEM; dynamic light scattering; FTIR; X-ray diffraction; induced coupled plasma optical emission spectroscopy; densitometry; micro-scratch tester; tribometer.	The tested materials presented relatively high Young’s modulus; the alumina hardness improved the abrasion resistance.
Wu et al. (2017) [36]	Particulate Science and Technology	10 for each composite	Laboratory study	To evaluate the effect of pH media (1,7,13) on mechanical and tribological properties: - Vickers microhardness (HV) - wear behavior as COF and wear rate of AP-X (Kuraray), Z350 (3M ESPE), Filtek P60 (3M ESPE), Vita Zeta (Vita), Vita LC (Vita) dental resin composites after 1, 13, and 20 days.	Vickers diamond indenter; optical microscopy; block-on-ring wear tester (lubricant used); SEM.	pH 1 and 13 induced greater damage on composites’ surface than neutral pH. Prolonged aging had a detrimental effect on mechanical and wear properties of composites. Under aging environment, nano-filled composites with low filler (VITA ZETA and VITA LC) exhibited worse mechanical and wear indices than the hybrid composites with high filler loading and distribution (AP-X, Z350 and Filtek P60).
Yadav & Meena (2021) [37]	Ceramics International	NR	Laboratory study	To determine the influence of a low amount (0, 2, 4, 6, and 8 wt%) of nHA and aluminium or titanium oxidum on the tribological (two-body sliding wear) behavior: wear rate of dental resin composites.	Pin-on- disc friction & wear test rig; SEM; EDS.	The composite with 0 wt% nHA at a normal load of 40 N and profile velocity of 140 RPM and 28 min exhibited the lowest wear rate for Al2O3-nHAmaterials. The composites with 8 wt% nHA at a normal load of 70 N, profile velocity of 110 RPM and 23 min reported the lowest wear rate. Overall, load, time, and filler significantly impacted the wear rate of tested composites.
Yadav et al. (2022) [38]	Polymer Composites	NR	Laboratory study	To assess the tribological behavior (two body sliding wear): wear rate of Ha-zinc oxide filled (from 0% to 8%) dental resin composites.	The pin-on-disc friction & wear test rig; SEM; EDS.	The tested composites with 8 wt% nHa at a load of 40 N, sliding speed 120 RPM 23 min showed a minimum wear rate. In general, load and filler significantly impacted the wear rate of tested composites.

AFM: atomic force microscopy; AR: acrylic resin; HA: hydroxyapatite; COF: coefficient of friction; EDS: energy-dispersive X-ray spectroscopy; FTIR: Fourier transform infrared spectroscopy; LED: light-emitting diode; NR: not reported; PE: polyethylene; PTFE: polytetrafluoroethylene; QTH: quartz–tungsten–halogen; SEM: scanning electron microscopy; SPM: scanning probe microscopy; TEM: transmission electron microscopy; WOF: work of fracture.3.2. Findings Summary.

## Data Availability

Data sharing not applicable–no new data generated.
